# Shadows Alter Facial Expressions of Noh Masks

**DOI:** 10.1371/journal.pone.0071389

**Published:** 2013-08-07

**Authors:** Nobuyuki Kawai, Hiromitsu Miyata, Ritsuko Nishimura, Kazuo Okanoya

**Affiliations:** 1 Okanoya Emotional Information Project (OEIP), Exploratory Research for Advanced Technology (ERATO), Japan Science and Technology Agency (JST), Nagoya, Japan; 2 Graduate School of Information Science, Nagoya University, Nagoya, Japan; 3 Okanoya Emotional Information Project (OEIP), Exploratory Research for Advanced Technology (ERATO), Japan Science and Technology Agency (JST), Wako, Japan; 4 Graduate School of Arts and Sciences, The University of Tokyo, Tokyo, Japan; New York University, United States of America

## Abstract

**Background:**

A Noh mask, worn by expert actors during performance on the Japanese traditional Noh drama, conveys various emotional expressions despite its fixed physical properties. How does the mask change its expressions? Shadows change subtly during the actual Noh drama, which plays a key role in creating elusive artistic enchantment. We here describe evidence from two experiments regarding how attached shadows of the Noh masks influence the observers’ recognition of the emotional expressions.

**Methodology/Principal Findings:**

In Experiment 1, neutral-faced Noh masks having the attached shadows of the happy/sad masks were recognized as bearing happy/sad expressions, respectively. This was true for all four types of masks each of which represented a character differing in sex and age, even though the original characteristics of the masks also greatly influenced the evaluation of emotions. Experiment 2 further revealed that frontal Noh mask images having shadows of upward/downward tilted masks were evaluated as sad/happy, respectively. This was consistent with outcomes from preceding studies using actually tilted Noh mask images.

**Conclusions/Significance:**

Results from the two experiments concur that purely manipulating attached shadows of the different types of Noh masks significantly alters the emotion recognition. These findings go in line with the mysterious facial expressions observed in Western paintings, such as the elusive qualities of Mona Lisa’s smile. They also agree with the aesthetic principle of Japanese traditional art “*yugen* (profound grace and subtlety)”, which highly appreciates subtle emotional expressions in the darkness.

## Introduction

Noh is a traditional form of Japanese musical drama originated in the 14th century (for an overview, see [1], [2]). As well as its length of history, Noh is regarded as a composite art that involves dance, drama, music, and so on, thereby well representing Japan’s cultural traditions. A Noh mask worn by skilled actors during performance is a hard wooden mask having fixed physical properties, which often looks expressionless at first glance. Nevertheless, during the actual Noh drama the mask conveys all kinds of different emotional facial expressions, as termed “*mugen hyojo* (infinite facial expressions)” [3], [4]. Specifically, a Noh mask can change its intended facial expressions according to various relevant factors including head/body orientation and breathing of the actor, background music, scenes of the drama, etc. Whereas complex, multisensory processing may potentially be involved in the appreciation of the Noh drama [4], it seems important to empirically examine potentially influential factors one by one, by which to specify which ones may play significant roles in characterizing the emotion recognition.

Using Noh masks as facial stimuli, Lyons et al. [5] showed that both British and Japanese participants attributed happiness to a downward tilted mask, and sadness to an upward tilted mask. Minoshita et al. [6] analyzed large numbers of emotions and found that, for example, downward tilted Noh masks were recognized as happier, more composed, and less surprised, than upward tilted masks (see also [7], [8]). Nishimura et al. [4] used images of either the Noh mask alone, or the mask together with the body, and revealed that pictures with small downward inclinations were recognized as sadder, while those with larger downward inclinations as happier. Miyata et al. [9] further demonstrated that different facial parts convey different emotions in a Noh mask, and that among the facial parts the mouth serves as a diagnostic feature in the categorical judgment of emotions. These preceding studies are in agreement that the vertical head/body orientation of the actor influences the recognition of emotions (see also [10]–[12]). The studies, however, revealed trends opposite to the convention of the Noh drama. That is, the mask is tilted upwards/downwards when signifying a happy/sad state, the gestures known as *terasu* (shining) and *kumorasu* (clouding), respectively [3]. To interpret these apparent contradictions, Nishimura et al. [4] proposed that multiple conventional styles of the Noh drama other than those regarding the masks themselves may be used for the appropriate appreciation of the intended emotions.

In the present study, we focus on the effect of the attached shadows resulting from the lighting of the stage, which is considered as one critical factor during the Noh drama. Literature involving adult humans has suggested that attached shadows (or shading) have compelling influence on the three-dimensional perception of shape [13], [14]. Sensitivity to shading information has also been shown in infants below one year of age [15], [16]. In the Western artistic paintings, shadow-related information such as luminance is suggested to often play a significant role in changing the emotional expressions of the characters [17], [18]. Using photographed facial images of adult humans, Oda [19] showed that both camera and light angles had significant effect on the impression of neutral faces. With lighting given from above or from the side, the faces tended to be evaluated as angrier and more disgusting, for example, than when the light was from the front. Regarding Noh masks, Suzuki [20] examined participants’ impressions of three types of masks with lighting from different directions. For the two types of Noh masks Zoonna and Fukai, the images having light from above/below tended to give positive/negative impressions, respectively. The other mask Chujo tended to consistently give negative impressions, as is the original characteristic of that mask.

To date, these has been no evidence on how purely manipulating attached shadows, instead of changing the lighting angles, may influence the facial expressions of the Noh masks. Nevertheless, it seems reasonable to assume that attached shadows are of vital importance in terms of subtly changing the observers’ impressions on the emotional expressions of the masks. That is, a Noh stage is characterized by its darkness, reflecting the lighting conditions of the ancient ages [21]. Small inclination of the actor’s head and the body during the drama conveys multiple subtle emotional changes [4], [9]. Lighting from above naturally causes many shadows at the lower half of a Noh mask, whereas lighting from below results in wide shadowed areas at its upper half. Further, with lighting from above, a downward tilted Noh mask is supposed to have more shadowed areas than a frontal mask with the same lighting. Consequently, it could be predicted that a Noh mask having many shadowed areas at its lower/upper half tend to be recognized as bearing happy/sad expressions, respectively. However, manipulating the directions of the light as implicated in preceding studies [19], [20] alters not only the shadows themselves but also the lit areas, or even the entire three-dimensional appearance of the face. To purely extract the effect of the shadows, it would be necessary to systematically manipulate shadow information, with no other changes such as the lighting directions to the original facial stimuli. In addition, it seems beneficial to examine multiple types of Noh masks differing in the represented sex and age, by which to evaluate the generalizability of the findings.

Accordingly, we here examined how attached shadows of the Noh masks influence the recognition of emotional expressions. Shadows of the happy/sad Noh masks produced by a computer graphic technique (Experiment 1) and those of an upward/downward tilted mask (Experiment 2) were overlaid to the neutral, frontal Noh mask images. Participants evaluated the emotions of these processed images as either happy or sad. Given the importance of the lighting conditions during the Noh drama, we expected that such addition of the shadows to the neutral Noh mask images would significantly influence the recognition of emotions.

## Results

### Experiment 1

**Figure 1:**
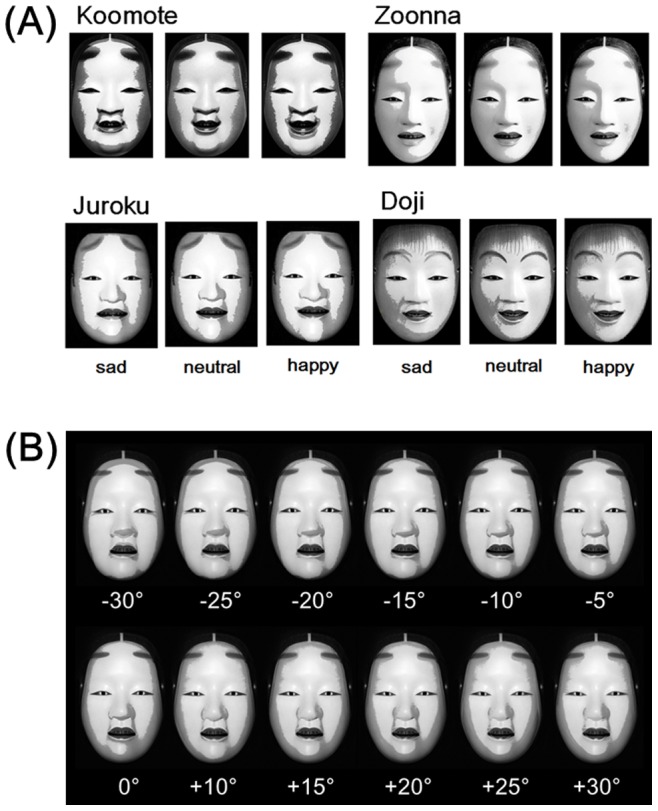
Noh mask images used as stimuli. (**A**) The 12 Noh mask images having overlaid shadows used in Experiment 1. Koomote, Zoonna, Juroku, and Doji each refers to the different type of the Noh mask. For each mask, there were three conditions regarding the emotional facial expressions: happy, neutral, and sad. (**B**) Test images used in Experiment 2, having shadows of the vertically tilted images overlaid to the frontal mask.

**Figure 2:**
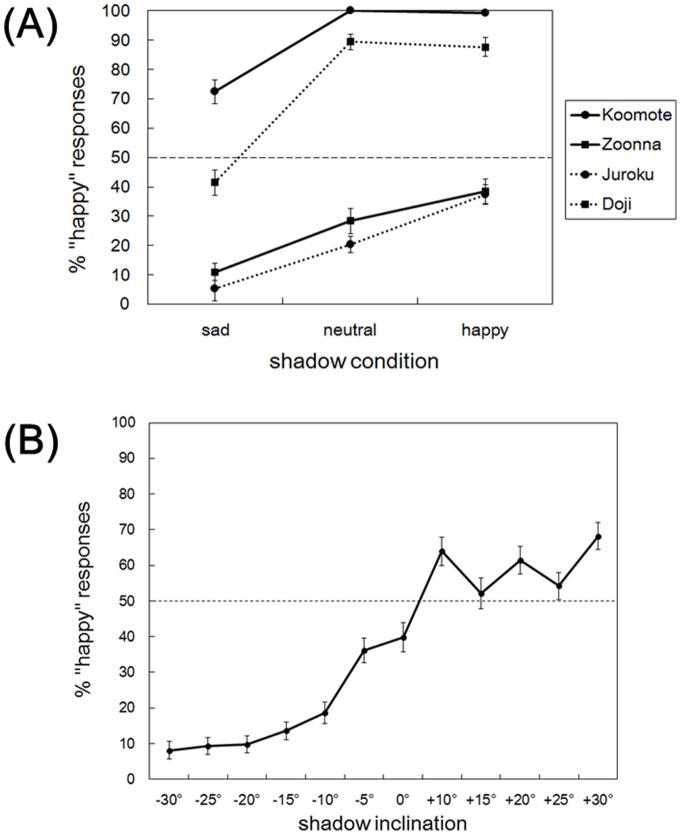
Results. (**A**) Proportion of the “happy” evaluation for each Noh mask type (i.e., Koomote, Zoonna, Juroku, and Doji) and shadow condition (i.e., sad, neutral, and happy) in Experiment 1. (**B**) Proportion of the “happy” evaluation for each shadow inclination in Experiment 2. The dashed horizontal lines indicate the chance proportion of the “happy” responses (50%). Error bars indicate standard errors of the mean.

For each shadowed image (**Figure 1**), the proportion of the “happy” evaluation was determined based on the number of times with the “happy” evaluation out of the four repeated trials. **Figure 2(A)** depicts these choice proportions for different Noh mask types and shadow conditions. Because the data points were given in the proportions, the data were transformed for the statistical analyses, using the equation *r’* = arcsin (√*r*), to make distributions more normal. For these choice proportions, a two-way repeated measures analysis of variance (ANOVA) with both Noh mask type (4 types: Koomote, Zoonna, Juroku, and Doji) and shadow condition (3 conditions: happy, neutral, and sad) as within-subject factors was conducted. The main effects of both Noh mask type (*F*(3, 174) = 191.57, *MSE* = 164338.13, *p*<0.001, η^2^ = 0.49) and shadow condition (*F*(2, 116) = 155.22, *MSE* = 57653.23, *p*<0.001, η^2^ = 0.11), and the interaction between these factors (*F*(6, 348) = 8.31, *MSE* = 2729.96, *p*<0.001, η^2^ = 0.02) all turned out to be statistically significant. Multiple comparisons with Bonferroni correction were then conducted for each Noh mask type. For Koomote, statistically significant differences were found between sad and neutral (*r* = 0.69, *p*<0.001), and sad and happy conditions (*r* = 0.68, *p*<0.001), though not between neutral and happy conditions (*p = *0.477). For Zoonna, there were significant differences between all condition pairs: sad and neutral (*r* = 0.54, *p*<0.001), sad and happy (*r* = 0.74, *p*<0.001), and neutral and happy (*r* = 0.32, *p = *0.035). For Juroku, significant differences were again found between all three condition pairs (*r = *0.49–0.63, all *ps* <0.001). For Doji, differences between sad and neutral (*r* = 0.81, *p*<0.001), and sad and happy conditions (*r* = 0.81, *p*<0.001) were statistically significant, though that between neutral and happy conditions were not (*p*>0.999). These results show that both different shadows and types of Noh masks significantly influenced the recognition of emotional expressions. Noh mask images having the overlaid shadows of sad and happy Noh-mask faces overall tended to be evaluated as sadder and happier than those having the shadows of neutral faces, respectively, even though the latter was not true for the two types of masks Koomote and Doji.

One-sample *t*-tests with Bonferroni correction further compared the proportion of “happy” evaluation for each combination of Noh mask type and shadow condition (i.e., Koomote-sad, etc.) with the chance level (50%). For Koomote-neutral, the proportion was 100%. For Koomote-sad, Koomote-happy, Doji-neutral, and Doji-happy, the proportions were significantly above chance level (*t* = 5.32–61.70, *r* = 0.57–0.99, all *ps* <0.001, corrected). For Zoonna-happy, Juroku-happy, and Doji-sad, the proportions did not significantly differ from chance (*t* = −2.61–−1.83, all *ps* >0.100, corrected). For Zoonna-sad, Zoonna-neutral, Juroku-sad, and Juroku-neutral, the proportions were significantly below chance (*t* = −22.80–−4.95, *r* = 0.55–.95, all *ps* <0.001, corrected). Thus, the judgment of emotions largely differed for each Noh mask type, such that Koomote and Doji overall tended to be evaluated as happy, and Zoonna and Juroku as sad.

### Experiment 2

One participant (male; 19 years) answered no questions and another (male; 18 years) failed to answer two of the questions. These data were excluded from analyses. For each shadow inclination (−30– +30 degrees), the proportion of the “happy” evaluation was determined based on the number of “happy” responses out of the four repetitions. **Figure 2(B)** depicts these proportions. As in Experiment 1, statistical analyses were conducted after arcsine transformation of the data. Data for +5 degrees were excluded due to errors in stimuli. Cochran-Armitage trend test revealed that a linear trend for these proportions was significant (*χ* ^2^ (1) = 9.47, *p = *0.002, η^2^ = 0.39), whereas the departures from linearity was not (*χ* ^2^ (10) = 0.94, *p*>0.999). Then, one-sample *t*-tests with Bonferroni correction revealed that, for −30 degrees, the proportion of “happy” evaluations was significantly lower than the chance level (50%) (*t*(58) = −15.63, *r* = 0.90, *p*<0.001, corrected). For +30 degrees, by contrast, the proportion was significantly higher than chance (*t*(58) = 4.53, *r* = 0.51, *p*<0.001, corrected). Thus, Noh mask images having shadows of upward tilted masks strongly tended to be evaluated as sad, and those with shadows of downward tilted masks as relatively happy.

## Discussion

### Experiment 1

The results revealed that neutral-faced Noh mask images having the attached shadows of a happy face tended to be evaluated as happier, whereas those with the shadows of a sad face as sadder, compared with the comparable images having shadows of a neutral Noh-mask face. This was true regardless of the fact that types of the Noh masks also had great impact on the proportions of the happy/sad evaluations. These data are thus supportive of the notion that shadows extracted from the emotional Noh-mask faces considerably alter the recognition of the emotional expressions. In addition, the effect of shadow conditions differed for different types of Noh masks, in that shadows extracted from the happy face failed to have significantly altered the emotion recognition for the two masks Koomote and Doji. These seem to result from a ceiling effect, because with neutral shadows these masks already had strong trends to be recognized as bearing happy expressions.

### Experiment 2

The data clearly demonstrated that the frontal images of the Noh masks having the attached shadows of the upward and downward tilted images of the same mask tended to be recognized as sad and happy respectively, showing a linear trend as the tilting angles increased. These results show trends opposite to the rules of Noh, consistently with the previous findings using vertically tilted Noh mask images [4]–[6], [9]. The present data thus suggest comparable effect of tilting angles, by simply manipulating the shadows of the Noh mask images. Specifically, the corners of the lips are pulled up for the downward tilted Noh mask, which are elements of a happy expression [5]. This seems to explain why downward tilted masks are recognized as happy (and vice versa), because the shape of the mouth serves as a distinctive feature in the judgment of emotions [9]. Further, the fact that the attached shadows of the smiling mouth made the mask look happy appears consistent with the smile of Mona Lisa, which is more apparent in the low spatial frequency [17]. Japanese observers are reported to gaze at the mouth less frequently than do Westerners, both when looking at emotional facial stimuli and emoticons [22]–[24]. In addition, the Noh stage is dark and full of shadows, and observers see the actors from a relatively distant location. Thus, during the actual Noh play, the shape of the mouth is not necessarily apparent from the observer’s viewpoint, where downward tilted masks may not be likely to look happy. However, when closely looking at the mask alone on a computer monitor, as done in the present study, the shape of the mouth should clearly serve as a distinctive feature in the recognition of emotions, resulting in downward tilted masks looking significantly happier than upward tilted ones [25].

## General Discussion

The present study examined how attached shadows of the Noh masks influence the recognition of emotions, by systematically overlaying shadows of emotional or tilted masks to the neutral, frontal images of the same masks. In Experiment 1, shadows extracted from happy and sad faces of the Noh masks significantly imposed happier and sadder impressions to the otherwise neutral-faced masks, respectively. Similarities and differences between four different types of masks are noteworthy, because a large part of the preceding studies involved only a single type of the mask [4]–[6]. The effect of shadows were overall similar for all types of Noh masks, even though the happy conditions regarding Koomote and Doji seem to have reflected a ceiling effect. This suggests generality of the effect of shadows across multiple types of masks differing in age, sex, and represented character. On the other hand, the proportions of “happy” evaluations considerably differed for each type of the Noh mask. Specifically, the two types of masks Koomote and Doji overall tended to be recognized as happy, while the others Zoonna and Juroku as sad, across the shadow conditions. Koomote, Doji, and Juroku all represent young characters below 20 years of age, while Zoonna portrays a relatively elderly woman. Koomote and Zoonna represent female characters, while Juroku and Doji do those of males. Thus, rather than age or sex of the characters, the original characteristics of each mask seem to have distinctively influenced the recognition of emotions. For example, whereas Juroku represents a young male, the character is thought to have been defeated and killed in a historical battle at the age of 16 [26], and may thus give a sad impression. It would be interesting to further examine whether comparable effects are observed for more extreme-faced Noh masks such as Hannya, which portrays a woman who has turned into a demon due to jealousy or obsession [26].

Experiment 2 further demonstrated that shadows extracted from vertically tilted images of a Zoonna mask cause changes to the recognition of emotions, in ways comparable to the images of actually tilted masks. As a whole, “sad” evaluations were more dominant (63.7% on the average) than “happy” ones. This may be because the overlaid shadows have imposed negative or dark impressions to the faces, as well as reflecting the original characteristics of the Zoonna mask, in the same ways as suggested in Experiment 1. Nevertheless, a clear linear trend regarding the increase in the proportion of “happy” evaluations was observed from upward to downward tilting angles. At the largest downward tilting angle (+30 degrees), the image was evaluated as happy significantly more often than chance. The results thus suggest the effect of shadows in terms of altering the emotional impressions of a Noh mask, by simply changing the head inclinations of the original images.

The effect of shadows as revealed in the present study is observed more widely across cultures. For example, in Beijing opera (Chinese classical opera) and Kabuki (Japanese classical drama), peculiar styles of makeup are painted on the actor’s face to emphasize the emotions of the character [27]. A number of tribes also have their own cosmetics to arrange emotional facial expressions. In the Western paintings, the elusive quality of the Mona Lisa’s smile is suggested to be more apparent with low spatial frequency, in which shadow information looks more emphasized than in high spatial frequency range [17]. Her smile thus is thought to be more apparent to peripheral vision than to central vision (see also [28]). Adding noise to the Mona Lisa images is also known to significantly alter facial expressions [29]. In the Noh drama, combinations of the vertical and horizontal movements and their moving speed are what an actor can control during the play. Subtle changes in the tilting angles create various facial expressions during the drama, in which shadow information should play a crucial role in conveying the character’s emotions. Junichiro Tanizaki, a renowned Japanese novelist, noted in his essay In Praise of Shadows that appreciation of the shadow and subtlety characterizes Asian culture [21]. As Tanizaki indicated, darkness of the Noh stage seems to enable artistic expressions of various emotions no less richly than do other dramas such as Kabuki. These also seem consistent with the concept of “*yugen* (profound grace and subtlety)”, which is regarded as the highest aesthetic principle in Noh [30]. Such traditional Japanese aesthetics seems to go in line with the enchantment of Mona Lisa. That is, low spatial frequency components of facial stimuli in the darkness may play a crucial role during the Noh drama in terms of conveying countless subtle and composite emotions, as referred to as “*mugen hyojo*” in the Introduction. Systematic control of spatial frequency for the Noh masks, as Livingstone [17] made for Mona Lisa, would promisingly verify these possibilities.

To summarize, the present study demonstrated that purely manipulating attached shadows of the different types of Noh masks alters the emotion recognition, overall in comparable ways as previous reports have suggested. A promising application of the present approach would be to actively utilize shadow information for manipulating the emotional facial expressions. Given the importance of shadows and darkness in the Noh drama, the effect of shadows as found in this study may be most apparent for the Noh masks. Nevertheless, comparable effect may be present in other dramas as well as in the actual social context. For example, in dramas using masks, the artists could actively emphasize shadows of specific facial parts to change emotional expressions. It may also be possible to manipulate shading information by using cosmetics, so that the human faces would give more positive impressions. These multiple approaches would be the milestones towards uncovering the mechanisms of emotion recognition by shadows and utilizing them in practical contexts.

## Materials and Methods

### Experiment 1

In Experiment 1, we examined whether adding the attached shadows of the emotional Noh-mask faces to the neutral-faced masks may influence the recognition of the emotional expressions. Shadows extracted from Noh masks with either happy or sad expressions were attached to the frontal, neutral images of the same masks. Assuming the significant effect of shadow-related information alone, we hypothesized that shadows of the happy/sad faces would impose happy/sad impressions to the neutral Noh masks, respectively. We used four different types of Noh masks, two representing females and the others representing males, to examine the potential differences in the recognition of emotions between these types.

#### Participants

Fifty-nine healthy Japanese adults (25 females and 34 males; mean age 19, range 18–20 years, *SD* = 0.5) participated. All participants in both experiments were familiar with Noh masks, either as images or objects, but had not received specialized training with Noh prior to participation. All participants had normal or corrected-to-normal vision. The study was approved by the Ethics Committee of the Japan Science and Technology Agency. All participants from both experiments gave written informed consent upon agreement cooperate.

#### Stimuli

Four different types of Noh masks, Koomote, Zoonna, Juroku, and Doji, were used (**Figure 1(A)**). Koomote and Zoonna are the masks classified into Onnamen (female mask), while Juroku and Doji are those called Otokomen (male mask). Specifically, Koomote is a mask representing a teenaged girl with serene beauty and attractiveness. Zoonna represents a woman who possesses nobility, decency, and intelligence. Juroku portrays a young male prince who was killed in a battle. Doji represents a young boy who is symbolizing the eternal youth as the incarnation of God [26]. All these masks had been carved by Akira Kurabayashi, a Japanese professional Noh mask artist. All the original images were the ones photographed from the front and had neutral facial expressions. The images were copy-free and were downloaded from the website of the artist [31]. The size of all the images presented during the tests was set at 11.4 degrees wide and 18.5 degrees long in terms of visual angle.

For each of the four types of Noh masks, images having happy and sad facial expressions were created by modifying the original neutral facial images [9]. FaceTool, a software for processing facial expressions, was used. The software had been developed by the laboratory of Professor Hiroshi Harashima, The University of Tokyo, Japan [32]. With FaceTool, it is possible to change the shapes of different facial parts, including the eyebrows, the eyes, the cheeks, the month, etc., to create various facial expressions. The software had 23 bars as controllers, each of which labeled “eyebrow: inner brow raise”, “lip: lower lip depress”, and so on, which were used to manipulate shapes of each facial part. These actions of facial parts corresponded to the Action Unit (AU) used in Facial Action Coding System (FACS) [33], a system that enables description of humans’ facial expressions based on the movements of mimetic muscles.

Then, shadows were extracted from the unprocessed, happy, and sad images of the four types of Noh masks. These and the later processes were implemented using Adobe Photoshop CS4. Specifically, the original colored images were first converted to grayscale. Then, only the facial areas for each image were selected, excluding the remaining areas including the background and the hair. Next, the average brightness for the facial areas of each image was calculated. Using this mean value as a threshold, each image was converted to black and white. Areas represented in black at this stage were operationally defined as the shadows for each image. These shadows extracted were then overlaid to the neutral, unprocessed images of each type of the Noh mask. Transparency of the layer for the shadows was set at 20%. These synthesized images were further converted to grayscale. Finally, brightness of the images was increased to 120 cd/m^2^, in order to make their appearance more natural. Trials with Noh mask images having the overlaid shadows of the unprocessed, happy, and sad faces are hereinafter referred to as neutral, happy, and sad conditions, respectively. These processes resulted in a total of 12 test images: 4 (Noh mask type: Koomote, Zoonna, Juroku, and Doji)×3 (shadow condition: happy, neutral, and sad), all of which are depicted in **Figure 1(A)**.

#### Procedure

The experiment took place in a room with a personal computer equipped for each participant. All participants were instructed and tested at a time. The test stimuli were presented on a 43 cm (17.0 inches) TFT LCD monitor (VL-17SE, Fujitsu, Kawasaki, Japan), with a screen resolution of 1280×1024 pixels. The monitor had a mean luminance of 250 cd/m^2^, and ran at a frame rate of 60 Hz. The monitor was located on a table in front of the participant, who sat on a comfortable chair. The distance between the monitor and the participant’s eyes was set at 50 cm. The test stimuli as well as the general instruction of the experiment were presented in slides of Microsoft PowerPoint 2007. Before the main test session, there were three practice trials using Noh mask images other than those for the test, for instruction of the general procedure. During the test session that followed, each of the 12 test images was presented four times, thereby 48 trials forming the session. The order of these images presented was pseudo-randomized. For each trial, the number of that trial first appeared for one second, followed by the test image for eight seconds at the center of the display on a white background. The duration of the presentation of stimuli was controlled by a personal computer, and the test displays switched automatically following the determined durations, regardless of the participants’ responses. Participants were informed during the instruction that they would not be allowed to terminate the session on the halfway. Participants answered whether each displayed Noh mask image expressed happy or sad emotions (using the Japanese terms “*yorokobi*” or “*kanashimi*”), by filling in the evaluation sheet placed on a table in front of them. The answer had to be given in the form of a two-alternative forced choice, and thus no answers in between were allowed. The duration of the test session was approximately seven minutes, and the whole experiment lasted for approximately 20 minutes.

### Experiment 2

It became evident from Experiment 1 that changing the shadow information influences the happy/sad impressions of the Noh masks. In the actual Noh drama, each mask harbors a single fixed facial expression, and the actors’ head/body orientation and other information convey the characters’ emotions. Previous studies are in agreement that Noh masks tilted upwards and downwards tend to be recognized as sad and happy, respectively [4], [5], [9]. Experiment 2 examined whether manipulating attached shadows alone may cause comparable changes in the recognition of emotions, by overlaying shadows of a Noh mask in different tilting angles to its frontal image.

#### Participants

Sixty healthy Japanese adults (22 females and 38 males; mean age 19, range 18–21 years, *SD* = 0.5), other than those included in Experiment 1, participated.

#### Stimuli

The original Noh mask used as stimuli was a Zoonna mask newly carved for the study in 2011 by Shigeharu Nagasawa, a Japanese professional Noh mask artist. The test stimuli were the images of this mask photographed at different vertical inclinations, from −30 to +30 degrees in equal 5-degree increments. The mask was photographed by a professional photographer from a frontal viewpoint, using a digital camera (Canon EOS 7D, Canon, Tokyo, Japan) with a 50 mm lens. The light source position was the same across images. Shadows were extracted and overlaid to the frontal image of the same mask, generally following the same processes as in Experiment 1. One difference was that the apparent two-dimensional size and shape of the face, as well as those of the shadows, varied for each inclination. Accordingly, the shadows were overlaid to the frontal images first by matching the positions of binocular pupils, then by extending the shadows equally in all directions so as to match the positions of the mouth. These test images are depicted in **Figure 1(B)**.

#### Procedure

The procedure was the same as in Experiment 1, except for the following changes. The test images were each presented four times during the test session, which consisted of 52 trials in total. The test session lasted for approximately eight minutes.
